# The prognostic potential and oncogenic effects of PRR11 expression in hilar cholangiocarcinoma

**DOI:** 10.18632/oncotarget.3983

**Published:** 2015-05-04

**Authors:** Ying Chen, Zhanshan Cha, Wenzheng Fang, Baohua Qian, Wenlong Yu, Wenfeng Li, Guanzhen Yu, Yong Gao

**Affiliations:** ^1^ Department of Pathology, Changhai Hospital, Shanghai, China; ^2^ Department of Transfusion, Changhai Hospital, Shanghai, China; ^3^ Department of Oncology, Fuzhou General Hospital, Fuzhou, Fujian Province, China; ^4^ Department of Surgery, Eastern Hepatobiliary Hospital, Shanghai, China; ^5^ Department of Radiation Oncology, First Affiliated Hospital of Wenzhou Medical College, Wenzhou, Zhejiang, China; ^6^ Department of Medical Oncology, Changzheng Hospital, Shanghai, China; ^7^ Department of Oncology, East Hospital, Tongji University School of Medicine, Shanghai, China

**Keywords:** PRR11, hilar cholangiocarcinoma, oncogene, prognosis

## Abstract

PRR11 is a newly identified oncogene in lung cancer, yet its role in others tumors remains unclear. Gastrointestinal tissue microarrays were used to evaluate PRR11 expression and its association with clinical outcome was analyzed in patients with hilar cholangiocarcinoma. Overexpression of PRR11 was observed in esophageal, gastric, pancreatic, colorectal, and hilar cholangiocarcinoma. Expression of PRR11 correlated with lymph node metastasis and CA199 level in two HC patient cohorts. After an R0 resection, a high level of PRR11 expression was found to be an independent indicator of recurrence (*P* = 0.001). In cell culture, PRR11 silencing resulted in decreased cellular proliferation, cell migration, tumor growth of QBC939 cells. Microarray analysis revealed that several genes involved in cell proliferation, cell adhesion, and cell migration were altered in PRR11-knockout cells, including: vimentin (VIM), Ubiquitin carboxyl-terminal hydrolase 1 (UCHL1), early growth response protein (EGR1), and System A amino acid transporter1 (SNAT1). Silencing PRR11 inhibited the expression of UCHL1, EGR1, and SNAT1 proteins, with immunoassays revealing a significant correlation among the levels of these four proteins. These results indicate that PRR11 is an independent prognostic indicator for patients with HC.

## INTRODUCTION

The molecular aberrations in the development of gastrointestinal tumors have been widely explored, and there a variety of candidate genes described that may help predict prognosis and act as therapeutic targets (HER2, EGFR, etc.). However, only a few of these biomarkers are routinely used in the clinic or investigated as part of clinical trials. This difference between the bench and the bedside has encouraged additional research efforts to identify effective genetic and molecular markers for the development and application of novel targeted therapies.

Dysregulation of the cell cycle components promotes tumor formation [1], and, therefore, the proteins controlling cell cycle progression are potential targets for anticancer strategies. Proline-rich Protein 11 (PRR11), a candidate oncogene, has been implicated in cell cycle progression and tumorigenesis of lung cancer. Silencing PRR11 induces S-phase arrest, inhibiting cell viability and tumorigenic potential [2]. Additionally, *in silico* analysis has suggested that high expression of PRR11 is significantly associated with poor prognosis in lung cancer patients [2]. However, the role of PRR11 expression in other tumors and its clinical relevance is not clear at present.

In the current study, immunohistochemical techniques were used to evaluate the expression of PRR11 in multiple gastrointestinal cancers. During this analysis, it was observed that PRR11 expression was increased in invasive hilar cholangiocarcinoma (HC) relative to normal tissue and precursor lesions. Therefore, the association of PRR11 and HC was investigated further to determine if the protein could be used as a predictor of prognosis in patients. PRR11 was silenced in a HC cell line in order to profile its biological role in the disease, and it was found that PRR11 knockdown results in decreased tumorigenicity. The potential mechanisms by which its knockdown resulted in anticancer effects were also investigated and discussed.

## RESULTS

### Expression profiles of PRR11 protein in tumors of digestive system

**Figure 1:**
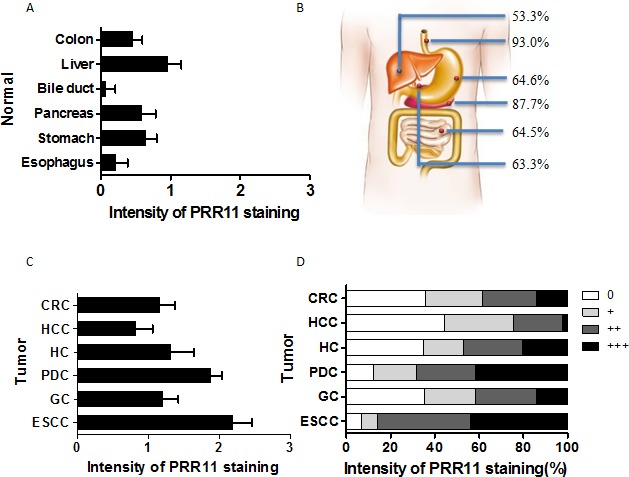
Expression patterns of PRR11 protein in 6 human gastrointestinal tissues and tumors **A.** The average intensity of PRR11 staining in normal gastrointestinal tissues; **B.** The rates of PRR11 positive staining in the 6 gastrointestinal tumors; **C.** The average level of PRR11 staining in these tumors; CRC, colorectal carcinoma; HCC, hepatocellular carcinoma; HC, hilar cholangiocarcinoma; PDC, pancreatic ductal carcinoma; GC, gastric carcinoma; ESCC, esophageal carcinoma; D, Distribution of PRR11 expression at different levels in these tumors.

PRR11 expression was evaluated in 6 different cancers of the digestive system. Differential expression of PRR11 was observed between tumor and normal tissue at various sites. A consistent low level of PRR11 positivity was observed in the epithelium of normal esophagus and bile ducts, and a high level of constitutive expression observed in liver tissue (Figure 1A). PRR11-positive expression was observed in 93.0% esophageal primary tumors, 64.6% gastric tumors, 64.5% colorectal tumors, 87.7% pancreatic ductal carcinomas, 53.3% hepatocellular carcinomas and 63.3% hilar cholangiocarcinoma (Figure 1B). Significant differences in PRR11 expression between normal tissues and tumors were observed in esophageal squamous cell cancer (ESCC), gastric cancer (GC), colorectal cancer (CRC), pancreatic ductal cancer (PDC), and hilar cholangiocarcinoma (HC), but not in hepatocellular carcinoma (HCC) (Figure S1). High expression of PRR11 was more common for ESCC, PDC, and HC (Figure 1C). Interestingly, the proportion of number of patients was uniformly distributed according to PRR11 staining level the three tumors (Figure 1D).

### PRR11 protein expression gradually increased along with progression of hilar cholangiocarcinoma

**Figure 2:**
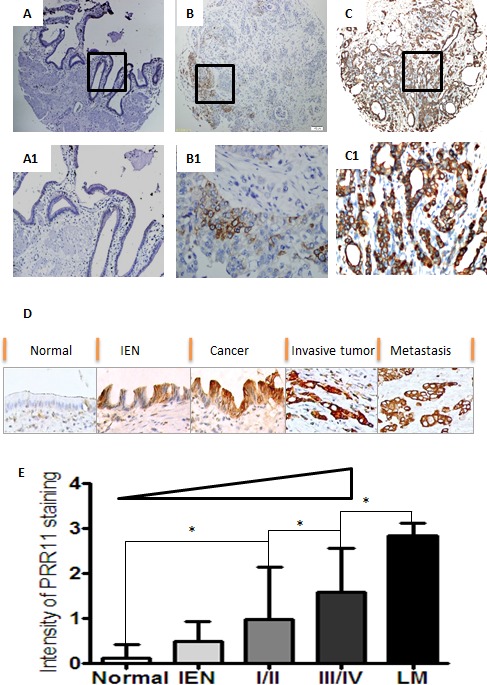
Expression profiles of PRR11 in hilar cholangiocarcinoma **A.** Normal bililary tissues with negative staining of PRR11; **B.** Tumor cells with weak staining of PRR11 (+); **C.** Tumor cell with strong staining of PRR11 (+++); **D.** A progressive tendency in the expression level of PRR11 from the precursor lesions to the metastatic lesions; IEN, intraepithelial neoplastic lesions; **E.** PRR11 expression correlates with disease progression. Original Magnification: A, B, C: IHC×40; A1, B1, C1, D: ×400; *P < 0.05.

PRR11 staining was predominantly cytoplasmic in neoplastic HC cells (Figure 2A-2D). The intensity of PRR11 staining increased with the progression of HC: 0.12±0.30 in normal bile duct tissues, 0.50±0.43 in intraepithelial neoplasia, 0.97±1.15 in stage I/II, 1.58±0.98 in stage III/IV and 2.83±0.28 in lymph node metastases (Figure 2D and 2E).

### Correlations between PRR11 expression and clinical variables

In the initial cohort, PRR11 expression was evaluated in 49 cases of HC. Most patients (93.9%) were at an advanced stage at the time of diagnosis, and, in 53.1% cases, radical excision could not be offered to the patient. PRR11 was positive by IHC in 31 (63.3%) of cases. There was a significant correlations between PRR11 expression and tumor invasion (*p* = 0.04) and lymph node metastasis (*p* = 0.048). At a median follow-up of 1.5 years (range, 0.1 to 4.92 years), 1.5-year OS rate in the discovery cohort was 35.5% and 66.7% for PRR11 positive and negative groups, respectively (Table 1, *p* = 0.010).

**Table 1:** Patient Characteristics by PRR11 Status

	Discovery Cohort		*P*	Validation Cohort		*P*
Parameters	N	n(%)		N	n(%)	
Age						
≤ 55y	25	15(60.0)	0.628	23	20(87.0)	0. 893
> 55y	24	16(66.7)		35	30(85.7)	
Gender						
Male	35	21(60.0)	0.453	40	35(87.5)	0.670
Femal	14	10(71.4)		18	15(83.3)	
Tumor size						
≤ 6cm	18	9(50.0)	0.142	27	21(77.8)	0.082
> 6cm	31	22(71.0)		31	29(93.5)	
T stage						
T1/T2	7	2(28.6)	0.04	46	39(84.8)	0.538
T3/T4	42	29(69.0)		12	11(91.7)	
N stage						
No	16	7(43.8)	0.048	30	22(73.3)	0.003
Yes	33	24(72.7)		28	28(100.0)	
Differentiation						
High/moderate	37	24(64.9)	0.683	54	47(87.0)	0.501
Poor	12	7(58.3)		4	3(75.0)	
Disease stage						
I/II	3	1(33.3)	0.267	24	17(70.8)	0.004
III/IV	46	30(65.2)		34	33(97.1)	
Positive margin						
No	23	14(60.9)	0.744	50	43(86.0)	0.909
Yes	26	17(65.4)		8	7(87.5)	
CA199						
Negative	10	4(40.0)	0.087	13*	9(69.2)	0.053
Positive	39	27(69.2)		43	39(90.7)	
CA199 levels						
Low	22	10(45.5)	0.020	27	21(77.8)	0.101
High	27	21(77.8)		29	27(93.1)	
Overall survival						
≤ 1.5 years	28	22(78.6)	0.010	29†	28(96.6)	0.019
> 1.5 years	21	9(42.9)		28	21(75.0)	

NOTE.*CA199 status was available for 56 cases in the validation cohorts. † One case was lost.

### Validation of prognostic value of PRR11 expression in patients with HC

To validate the association between PRR11 expression and malignant behavior of HC, we performed IHC of PRR11 in a separate cohort of 58 patients with HC with recorded clinical data. In contrast to the initial group, most patients (86.2%) underwent radical excision of the tumor and 41.4% presented at early stage by the time of diagnosis. The rate of PRR11 positivity was 86.2%. Significant correlations were observed between PRR11 positivity and lymph node metastasis and advanced disease stage. The OS of this cohort was similar to our initial cohort. At a median follow-up of 1.5 years (range, 0.3 to 4.0 years), 1.5-year OS rate in the validation cohort was 54.0% and 87.5% for PRR11 positive and negative group, respectively (Table 1, *p* = 0.019).

### Correlations between PRR11 expression and CA199 level

Serum tumor marker levels, specifically CA199, were associated with tumor stage, tumor recurrence, and worse overall survival [3]. The expression of PRR11 was correlated with serum CA199 levels in both cohorts and the overall cohort. In accordance with clinical criteria, CA199 levels were defined as either negative or positive. Based on a median level of CA199 of 244 U/ml (range, 2.1 to >1000), CA199 level as either low or high. In the cohorts, significant correlations were observed between CA199 levels and disease-free survival and overall survival (Figure S2A and B). There was a strong correlation between PRR11 expression and CA199 levels in both cohorts (Table 1) (Figure S2C and D). Moreover, PRR11 expression was significantly associated with both CA199 status (*r* = 0.230, *p* = 0.018) and CA199 levels (*r* = 0.259, *p* = 0.008) in the overall cohort (Table S1).

### PRR11 overexpression is associated with disease-free survival in HC with R0 resection

**Figure 3:**
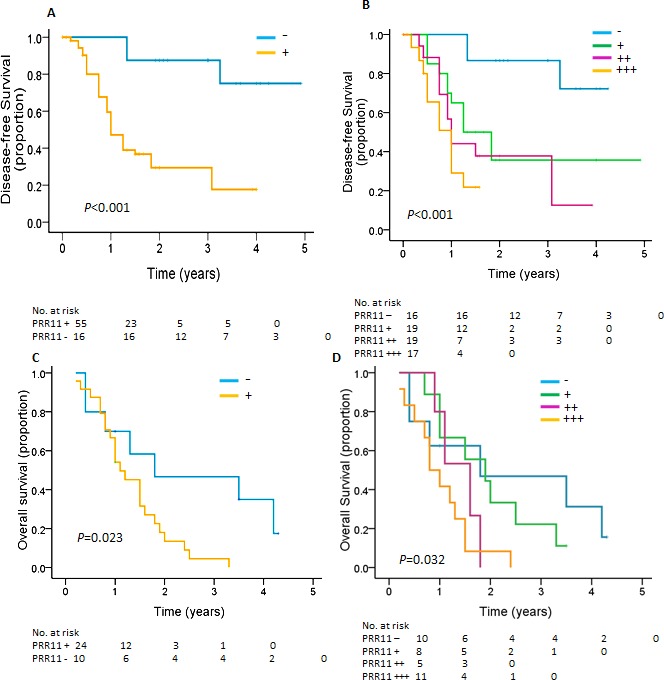
Kaplan-Meier estimates of disease-free survival and overall survival for HC patients according to PRR11 **A. B.** Kaplan-Meier curves for disease-free survival for patients with R0 resections are shown according to expression of **A.** PRR11 status and **B.** PRR11 expression levels. C, D, Kaplan-Meier curves for overall survival for patients with R1/R2 resections are shown according to expression of **C.** PRR11 status and **D.** PRR11 expression levels.

The prognostic significance of PRR11 was determined for the combination of the two cohorts. Among 107 HC patients, 68.2 %( 73/107) underwent R0 resection. After an R0 resection, 38 patients experienced recurrence at a median of 0.92 years (range, 0.17 to 3.25 years), 46 patients died of disease at a median of 1.33 years (range, 0.42 to 3.83), 25 patients are alive without recurrence, and 2 patients were lost to follow-up. In a univariate analysis, PRR11 status, tumor size, tumor invasion, lymph node metastasis and disease stage, were significant predictors of tumor recurrence. Patients with PRR11-positive tumors had inferior time to recurrence compared to that of PRR11-negative tumor (*p* < 0.001, Figure 3A). Multivariable analysis showed that PRR11positivity, rather than other clinical variables, was an independent predictor of tumor recurrence (Table 2). We also examined the association between the different levels of PRR11 expression and time to recurrence of HC patients. As expected, patients with PRR11- negative tumor had a more favorable time to recurrence than those with any level of PRR11 positive tumor (*p* = 0.002, *vs.* PRR11+ tumor; *p* < 0.001, *vs.* PRR11 ++ and +++ tumor, Figure 3B). Interestingly, PRR11 +++ patients showed an extremely unfavorable prognosis (median time to recurrence: 0.897 years) compared with PRR11 + patients (median time to recurrence: 1.25 years, *P* = 0.063) and PRR11 negative patients (median time to recurrence: 4.26 years, *p* < 0.001). No significant difference was observed between PRR11 ++ and +++ patients.

**Table 2:** Univariate and Multivariate Analysis of Variables Associated With DFS in Patients Submitted to an R0 Resection

		Median Survival (years)					
Variable	No. of Patients	Median	Range	*P* (univariate)	*P* (multivariate)	Hazard Ratio	95% CI
Tumor size							
≤ 3cm	33	> 3.20	0.08–4.92	0.028	0.732	0.864	0.373 to 1.998
> 3cm	38	1.25	0.17–4.25				
Tumor stage							
T1/T2	43	> 3.04	0.17–4.92	0.032	0.200	0.569	0.240 to 1.348
T3/T4	28	1.25	0.08–4.25				
Regional lymph nodes positive							
No	51	3.08	0.17–4.92	0.072	0.702	0.857	0.388 to 1.892
Yes	20	1.00	0.08–4.00				
TNM stage							
I/II	25	> 3.36	0.17–4.92	0.028	0.830	0.896	0.330 to 2.436
III/IV	46	1.25	0.08–4.25				
PRR11							
Negative	16	> 4.26	0.08–4.92	< 0.001	0.001	0.129	0.037 to 0.446
Positive	55	1.71	0.08–4.00				
CA199 levels							
Low	16	1.83	0.42–4.92	0.054	0.420	0.759	0.388 to 1.483
High	55	1.00	0.08–4.00				

### PRR11 overexpression is associated with decreased overall survival in HC patients undergoing R1/R2 resection

Among 107 patients, 31.8 %(34/107) received R1 (20, 58.8%) or R2 (14, 41.2%) resection. At a median OS of 1.13 years, 30 patients died due to their disease and 4 patients were still alive without recurrence. Patients with PRR11-positive cancer had a shorter OS than those with PRR11-negative tumor (1.1 years *vs.* 1.8years; *P* = 0.023, Figure 3C) (Table S2). However, no difference was observed between PRR11-negative patients and PRR11 + and ++ patients and between PRR11 ++ patients and PRR11 +++ patients. Interestingly, PRR11 +++ patients showed an extremely unfavorable prognosis (median OS: 0.80 years) compared with PRR11 - patients (median OS: 1.80 years, *P* = 0.021) and PRR11 + patients (median OS: 4.262 years, *P* = 0.049) (Figure 3D). None of the clinical variables was associated with OS of HC patients.

### Knocking down PRR11 expression inhibits cell proliferation and migration of HC cells

**Figure 4:**
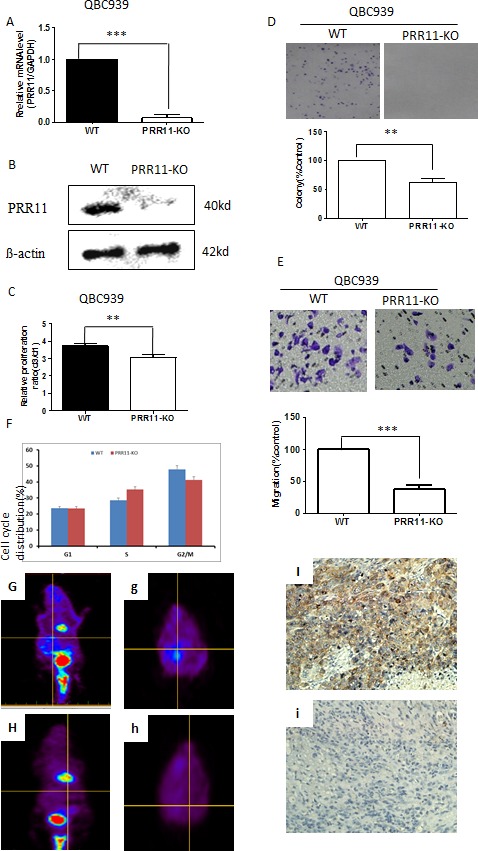
PRR11 knockdown inhibits cell proliferation and cell migration *in vitro* and *in vivo* **A.** The mRNA level of PRR11 in PRR11-siRNA (KO) and empty vector (WT) transfected cells. **B.** Levels of PRR11 protein in WT and PRR11-KO groups. **C.** CCK8 assay demonstrates the ratio of cellular proliferation in two groups. **D.** Colony formation assays in WT and PRR11-KO groups. **E.** Transwell assay demonstrating the effects of PRR11-knockdown on cell migration. **F.** Flow cytometric analysis demonstrating the cell cycle distributions of the two cell lines. **P < 0.01, ***P < 0.001 compared to the control. **G. H.** PET scan showed tumor location, distribution, and tumor size in the control group (G, g) and in the PRR11-KO group (H, h). (I, i) IHC showed strong expression of PRR11 in resected tumors from the WT group **I.** and negative staining of PRR11 in resected tumors from the PRR11-KO group (i). IHC×400.

To confirm the pro-tumorigenic role of PRR11 in hilar cholangiocarcinoma, QBC939 cells were transfected with PRR11-shRNA for stable knockdown (PRR11-KO) and compared to control. Infection with the PRR11-shRNA was accompanied by a decreased level of PRR11 mRNA (Figure 4A) and protein (Figure 4B). The ratio of cell proliferation was significantly reduced in PRR11-shRNA transfected cells compared with control as revealed by CCK8 assay (Figure 4C) and colony formation assay (Figure 4D), as well as cell migration (Figure 4E). While transient overexpression of ectogenic PRR11 in PRR11-shRNA stably infected cells slightly reversed the inhibition of cell proliferation (Figure S3). Additionally, cell cycle analysis showed an increased proportion cells arrested at S phase (Figure 4F).

### PRR11 depletion leads to inhibition of tumor growth of HC cells

To examine whether PRR11 downregulation could inhibit tumor growth *in vivo*, PRR11-KO and control cells were injected into the hepatic hilar area of BALB/C-nu mice. PET scans conducted on the mice demonstrated multiple lesions in the abdomens of the control mice which were not observed in the PRR11 knockdown mice (Figure 4G and 4H). Silencing of PRR11 expression (Figure 4I and 4i) significantly inhibited tumor growth as determined by both tumor weight and tumor size, as well as the expression of proliferation related indicators (Ki67), in the PRR11-KO inoculated mice compared with the control group (Figure S4).

### cDNA array and analysis

**Figure 5:**
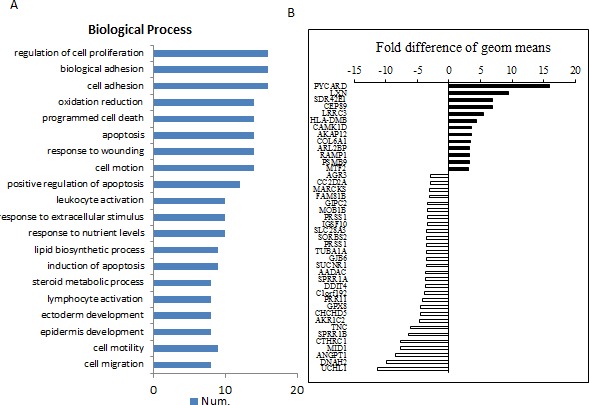
Differential gene expression patterns between PRR11-shRNA and empty vector transfected cells QBC939 cells were stably transfected with PRR11-shRNA or empty vector and subjected to microarray analysis. **A.** The most valuable biological processes of selected genes determined by Gene ontology analysis (*p* < 0.01, *n* ≥8). **B.** The selective top changed genes according to the order determined by the ratio of the two groups.

Since silencing PRR11 lead to marked inhibition of tumor cell growth and increased survival, the transcriptional effects of PRR11 expression was probed using cDNA array, specifically by comparing the gene expression patterns between PRR11-KO and wild-type cells. A total of 80 genes were found to be up-regulated, and 213 genes were down-regulated in the PRR11-KOcompared to control. A number of genes were found to be significantly altered as determined by Gene ontology analysis (Figure 5A, *p* < 0.01, *n*≥8). Differentially-expressed genes were grouped according to the ratio of expression between the two groups (Figure 5B). The correlation between the changed genes and the biological processes was determined by go-network analysis (Figure S5).

### PRR11 knocking down induces EMT

**Figure 6:**
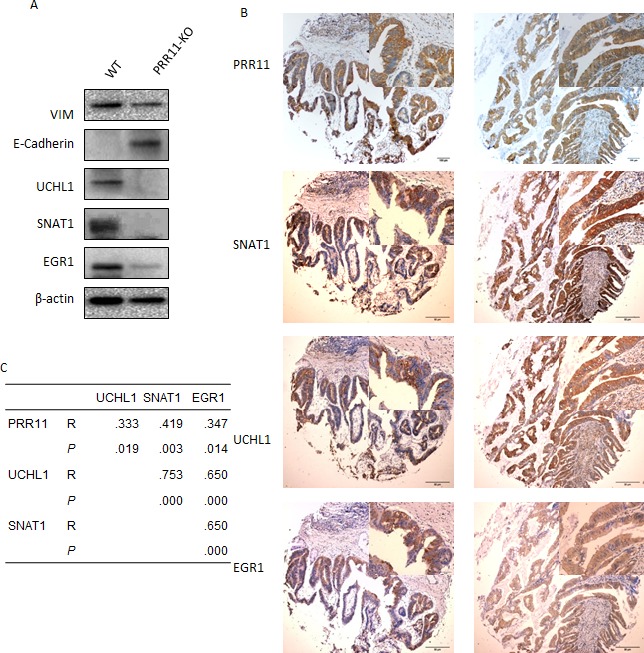
Validation of selected genes in HC cells and tissue samples **A.** The lysates of QBC939 cells transefected with PRR11-shRNA or empty vector were probed for expression of Vim, E-Cadherin, UCHL1, SNAT1, and EGR1 proteins by Western blotting. **B.** Positive expression of PRR11, UCHL1, SNAT1, and EGR1 proteins in human hilar cholangiocarcinoma as determined by immunohistochemistry. Original Magnification: Large pictures: IHC×40; Small pictures: ×400. **C.** Correlation among PRR11, UCHL1, SNAT1, and EGR1.

Among these biological processes, genes associated with cell adhesion and cell migration were mostly altered in PRR11-KO cells (e.g. VIM, LAMA3, LAMC2, and ITGAV). As VIM is a marker of epithelial-mesenchymal transition (EMT), the effect of PRR11 on cell migration through EMT was investigated. Knockdown of PRR11led to decreased expression of VIM protein and increased expression of E-cadherin (Figure 6A).

### PRR11 knocking down inhibits UCHL1, SNAT1, and EGR1 expression

PRR11 potentially participates in multiple biological functions as determined by the above cDNA analysis (Figure 5), and the function of PRR11 on tumor growth probably depends on the actions of downstream regulatory proteins. Several candidate gene products (UCHL1, SNAT1, and EGR1) were selected to investigate whether PRR11 influences their expression in HC cancer cells. Down-regulation of PRR11 led to decreased expression of UCHL1, SNAT1, and EGR1 proteins *in vitro* (Figure 6A) and *in vivo* (Figure S4E). Expression of UCHL1, SNAT1, and EGR1 proteins was predominantly localized to the cytoplasm and was observed in 55.1% (27/49), 55.1% (27/49), and 44.9% (22/49) of hilar cholangiocarcinoma samples, respectively (Figure S6 and Table S3). Expression of PRR11, UCHL1, SNAT1, and EGR1 was also found to positively correlate in most other types of cancer (Figure 6B and 6C) Moreover, SNAT1 and EGR1 expression was significantly associated with regional lymph node metastasis, and a trend was observed between UCHL1 expression and lymph node metastases (Table S3, *p* = 0.078). Survival analysis revealed that SNAT1 is an important predictor for tumor recurrence and prognostic factor for HC patients, while UCHL1 and EGR1 was not a significant indicator of prognosis (Figure S7).

## DISCUSSION

Alteration of cell cycle regulatory proteins is an essential step in carcinogenesis [1]. PRR11 is a newly identified gene that is located on chromosome17q22. The PRR11 gene product contains multiple highly conserved motifs in the C-terminus that are associated with cell cycle regulated protein degradation. In prior studies, PRR11 was suggested to be a negative effector in cell cycle regulation of tumor cells [2]. However, little is known about its distributions and clinical value in human solid tumors. In the current study, immunohistochemical techniques were used to assess expression levels of PRR11 in six gastrointestinal tumor types and the expression of PRR11 was correlated with clinical outcomes in patients with HC. We also explored the possible mechanism underlying PRR11 promoting cell proliferation and tumor growth of HC.

The expression profile of PRR11 has been investigated in lung cancer, and the protein appears tobe involved in progression of the cell cycle. PRR11 was found to be over-expressed in about half lung cancer specimens [2]. In the present study, we found that PRR11 was broadly expressed in human solid cancers, with the highest expression in ESCC (93.0%) and lowest levels in HCC (53.3%). This result confirmed a prior observation that PRR11 was specifically up-regulated in squamous cell carcinoma of lung cancer [2]. Expression of PRR11 was significantly altered in 5 tumors of digestive system (ESCC, GC, PDC, CRC, and HC), but not in HCC. The higher rates of PRR11 staining in ESCC and PDC will render PRR11 not suitable for prognostic study and the genetic and molecular pathogenesis of HC is currently unclear, therefore the current study was designed to explore the clinical significance of PRR11 in HC.

Hilar cholangiocarcinoma (HC) is a rare disease with aggressive behavior and an extremely poor prognosis [4]. Complete resection (R0) of the tumor offers the only chance for long-term survival. It was well-known that lymph node metastases, tumor grade and negative margins are important indicators for predicting tumor recurrence and overall survival [5]. We confirmed that lymph node metastasis, tumor invasion, and disease stage were associated tumor recurrence. However, none of these clinical variables were found to be independent predictors of tumor recurrence and patient outcome. In a prior study, staging did not correlate with survival after resection of hilar cholangiocarcinoma [6, 7]. A minimal sample size in the current study precluded the generation a more effective staging system.

Tumor markers, especially when combined with other diagnostic modalities, have been used for the diagnosis, treatment and monitoring of HC [3]. CA199 has been widely used to monitor patients for tumor recurrence and can predict a worse overall survival for tumors of digestive systems, particularly in pancreatic cancer and cholangiocarcinoma [3, 8]. Consistent with prior observations, serum CA199 levels were found to correlate significantly with frequent recurrence and short survival of HC patients. Interestingly, expression of PRR11 was found to be associated with a higher level of serum CA199.

PRR11 expression, as determined by IHC, is elevated in HC cells compared with normal tissues; however, inflammatory bile duct tissues and biliary intraepithelial neoplastic lesions (IEN) showed relative weak staining of PRR11. Moreover, expression of PRR11 in cancer cells in invasive and metastatic areas was much more intense than normal biliary epithelium and low grade IEN. Therefore, a progressive tendency in the expression pattern of PRR11 could be observed from the precursor lesions to the metastatic lesions. These data suggest a crucial role for PRR11 in the initiation and progression of HC.

Overexpression of PRR11 in HC tumor cells may predict a more aggressive clinical behavior. Several associations between PRR11 immunohistochemical staining and histopathological variables were observed in HC, mostly notably with nodal metastasis [9]. Furthermore, there was a significant correlation between high expression of PRR11 and clinical outcome. Expression of PRR11 was associated with shorter time to recurrence and OS in the combined cohort. Focusing our analysis, a shorter time to recurrence in patients with R0 resections or shorter OS for patients with R1/R2 resections was found for patients with high tumor expression of PRR11. By stratifying the expression intensity of PRR11, the highest level of PRR11 expression was demonstrated to predict recurrence and profoundly worse clinical outcomes in patients of HC. These data suggest that the utility of PRR11 should be investigated in prospective cohorts in future studies.

Although the current study sheds light on the pattern of PRR11 expression in six gastrointestinal tumors and potential clinical significance of PRR11 in HC, the potential functions and underlying mechanisms of PRR11 overexpression remain unclear. In lung cancer, silencing PRR11 induced S-phase arrest, inhibition of cell proliferation and invasion and especially tumor growth [2]. PRR11 knockdown leads to dysregulation of several pathways and genes involved in cell cycle and tumorigenesis. In present study, it was confirmed that PRR11-KO inhibited cell proliferation and migration, as well as induced S-phase arrest, *in vitro* and transplanted tumor growth *in vivo*. Moreover, we found that genes related with cell proliferation, cell adhesion, cell motion, cell motility, and cell migration were significantly altered following PRR11 knockdown, implicating a critical role of PRR11 in tumor progression. Expression of vimentin (VIM) mRNA and protein was also down-regulated when PRR11 was silenced, indicating a potential role of PRR11 in the initiation of EMT (which is supported by the finding that knockdown increased expression of E-cadherin). Taken together, the current findings support an oncogenic role for PRR11 in cholangiocarcinoma.

De-differentiation and abnormal metabolism are key characteristics of malignant tumor cells. We selected two differentiation-related genes (UCHL1 and EGR1) [10, 11] and one metabolic-related gene (SLC38A1/SNAT1) for further validation and correlation study. Ubiquitin carboxyl-terminal hydrolase 1 (UCHL1) belongs to the family of de-ubiquitinating enzymes (DUBs), deregulation of which causes tumor- inhibiting or -promoting functions in human cancer cells [12, 13]. Either up-regulation or down-regulation of UCHL1 has been observed in cholangiocarcinoma [10, 14]. In the present experiments, UCHL1 expression was absent/weak in normal cells, but highly expressed in tumor cells. In addition, UCHL1 expression was more frequently observed in patients with regional lymph node metastasis, implicating UCHL1 acting as a cholangiocarcinoma oncogene. Early growth response-1 (Egr-1) directs tendon differentiation [15] and regulates cell growth, proliferation, differentiation and apoptosis. Interestingly, EGR1 is also considered to be either a tumor-suppressor or tumor-promoter in various cell types [16, 17]. In present study, EGR1 was highly expressed in HC samples and correlated significantly with lymph node metastasis, implicating the pro-tumorigenic role of EGR1 in HC. The transport of glutamine through System ASC has been shown to associated with hepatocellular transformation [18], and increased expression of SNAT1 was previously shown to be aninverse predictor of prognosis in HC patients [19]. The above data confirm that SNAT1 is overexpressed in HC and is significantly associated with lymph node metastasis, tumor recurrence and poor survival. In addition to reduced expression of UCHL1, EGR1, and SNAT1 mRNA, silencing PRR11 diminished their protein levels by Western blot analysis. Co-expression of PRR11, UCHL1, EGR1, and SNAT1 was found to be a frequent event in HC tumor samples, indicating a cross-talk among these four proteins. Taken together, PRR11 may be involved cellular differentiation, activation, and amino acid transport by regulating UCHL1, EGR1, and SNAT1 expression. However, further studies are needed to highlight the exact mechanisms underlying their cross-talk promoting carcinogenesis and tumor progression.

In summary, this study demonstrates that PRR11 is widely up-regulated in human gastrointestinal tumors, including HC. Most importantly, the level of PRR11 expression is associated with adverse clinical outcomes in patients with HC. Targeting PRR11 caused inhibition of cell growth and tumor survival by reversing EMT and inhibiting activity of key regulators involved in cell migration and invasion. These findings strongly support an oncogenic role for PRR11 in HC and suggest that inhibition of PRR11 may be a future therapeutic target in the treatment of HC patients.

## MATERIALS AND METHODS

### Cohort selection for gastrointestinal cancer patients

The study group consisted of patients with esophageal squamous cell cancer (ESCC, *n* = 43), hepatocellular carcinoma (HCC, *n* = 45), pancreatic ductal cancer (PDC, *n* = 163), colorectal cancer (CRC, *n* = 93), gastric cancer (GC, *n* = 79), and hilar cholangiocarcinoma (HC, *n* = 49). These tumor and patient data were collected from the Changhai Hospital (ESCC, HCC, PDC, and CRC), Changzheng Hospital (GC) and Eastern Hepatobiliary Hospital (HC). All patients recruited to the study were approved by an institutional review board at their corresponding institutions. For the 49 patients with HC, clinical, recurrence and survival data were provided for analysis by the Eastern Hepatobiliary Hospital during 2005-2007. To validate data and statistics from this cohort, additional observational data was collected from an independent cohort of 58 patients with HC obtained from Changhai Hospital and Eastern Hepatobiliary Hospital during 2008-2010. All these patients were available for follow-up. The follow up endpoint was 2013.06. Histologic parameters and diagnoses were re-evaluated for this study by slide review. Tumors were re-staged according to the American Joint Committee on Cancer (AJCC) Staging Manual (seventh edition). The detailed clinical information from both cohorts is listed in Table 1, including recurrence and survival data.

### Tissue microarray

Tissue microarray (TMA) was constructed as previously described [20]. Briefly, hematoxylin and eosin-stained slides were evaluated, and representative malignant and adjacent non-neoplastic tissues were marked on the slides and then on the corresponding paraffin blocks. Two representative 1.5-mm-diameter tissue cores were taken from the donor samples and embedded into a recipient block using a tissue-array instrument (Beecher Instruments, Silver Spring, MD). Thirteen TMA blocks were designed containing samples from 472 patients (1 for ESCC, 1 for HCC, 3 for PDC, 2 for CRC, 2 for GC, and 4 for HC).

### IHC staining

Consecutive sections (4-μm) of tissue microarray samples were mounted on the APES-coated slides. After de-paraffinization, rehydration and antigen retrieval using an autoclave-oven-technique, sections were incubated with anti-PRR11 (HPA023923, SIGMA-ALDRICH), UCHL1 (HPA005993, SIGMA-ALDRICH), SNAT1 (ab59721, abcam®), and EGR1 (ab54966, abcam®) at 4°C overnight. A two-step EnVisionTM KIT (DAKO, USA) was used to visualize positive staining. Lung cancers known to be positive for these proteins were used as positive controls. Replacement of the primary antibody by PBS served as a negative control.

### Evaluation of staining of tissue slides

PRR11 expression in these 472 cases was evaluated by two individuals using an Olympus CX31 microscope (Olympus Optical). A semi-quantitative scoring system was used. In brief, staining intensity was assigned as follows: negative, 0; weak, 1; moderate, 2; and intense, 3. The percentage of immunoreactive cells was scored as 0∼1(0%∼100%). Theoretically, a weighted score ranging from 0 (0% of cells staining) to 3 (100% of the cells staining at 3+ intensity) was generated for each tissue core [20]. The final score for each tumor was calculated by averaging the score for two tissue cores. The case score > 0 was considered as positive. A four-tiered scale [0∼0.5, negative; 0.5∼1.5, weak (+); 1.5∼2.5, moderate (++); ≥;2.5, intense (+++)] was generated to grade the staining level of PRR11 for each case.

### PRR11 knockdown

Lentivirus expression plasmid containing small interference RNA (5′-ACGCAGGCCUUAAGGAGAATT-3′) targeting PRR11 was constructed by GENECHEM Corporation (Shanghai, China) and were transfected into cancer cells. Generally, 5 × 10^4^ QBC939 cells were planted in 6-well plate and lentivirus containing PRR11-RNAi and control was added into the supernatant.

### Real-time RT-PCR

Real-time RT-PCR of PRR11 in tumor cells was carried out using by SYBR Premix Ex Taq (Perfect real-time) kit (Takara) in a Rotor Gene 3000 system (Corbet Research, Sydney, Australia). GAPDH was used as the internal control. Relative mRNA abundance was calculated as PRR11/GAPDH. The primers used for PRR11 are as follow: forward: 5′- CGTATCTGCCACCGAGAACTT-3′, reverse: 5′- GAGATGGTCTTCAGTGCTTCCT-3′; GAPDH: forward: 5′-TGACTTCAACAGCGACACCCA-3′, reverse: 5′- CACCCTGTTGCTGTAGCCAAA-3.

### Cell proliferation assay

Cells with stably-transfected PRR11-siRNA (PRR11-KO) or empty vector (control) were digested and seeded in 96-well plates at a density of 5,000 cells per well. CCK8 assay (Dojindo Kumamoto, Japan) was performed to measure the final results at 24h and 48h. The proliferation ratio was calculated as the absorbance at 48h compared with that at 24h.

### Colony formation assay

PRR11-KO and control cells were plated in 6-well plates at a density of 2,000 cells per well and cultured for 2 weeks after infection. The cells were fixed in 100% alcohol and stained with crystal violet. Colonies with more than 50 cells per colony were counted.

### Transwell assay

PRR11-KO cells and cells transfected with empty vector were collected and resuspended in serum-free media at a density of 1 × 10^5^ cells/ml. 200μl cell suspension was introduced into the top chamber of the transwell, while 0.5ml DMEM with 5%FBS was placed into the lower chamber. After 24h incubation, the filters were taken out, fixed with methanol for 10min, and stained with 0.5% crystal violet reagent for 15min. The number of migrated cells on the lower side of the filter was then determined.

### Western blot analysis

Whole-cell lysates were prepared from stably PRR11-shRNA or empty vector transfected cells. Cell lysates were resolved by SDS/PAGE and transferred electrophoretically to PVDF membrane (Bio-Rad Lab., Hercules, CA, USA). The membranes were probed with specific antibodies, which were detected using an enhanced chemiluminescence (ECL) kit (Santa Cruz, CA, USA) [21].

### Flow cytometric analysis

Flow cytometric analysis was performed as described previously to determine the effects of PRR11 knocking down on cell cycle distribution [2]. Briefly, gastric cells were harvested by trypsinization and fixed with 70% ethanol, and measured according to the manufacturer's protocol (KEY GEN, Nanjing, China). Cell cycle distribution was analyzed by flow cytometry (FACSCalibur, BD Biosciences, Bedford, MA).

### Animal models

*In situ* tumor transplantation of hilar cholangiocarcinoma was carried out by inoculating BALB/C-nu mice (male; age: 4 weeks; 5/group) with cell suspensions (1*10^7^ QBC939 WT or PRR-11-KO cells) injected into the hepatic hilar area. Animals were housed under specific pathogen-free conditions and sacrificed 3 weeks after surgery. Animal PET was used to determine tumor location and size. All experimental manipulations were performed in accordance with the NIH Guide for the Care and Use of Laboratory Animals and approved by the Biomedical Ethics Committee of the Second Military Medical University (Shanghai, China).

### cDNA array

Total mRNA of control and experimental cells were collected for cDNA microarray analysis. Gene expression profiling was performed using Affymetrix® Human Genome U219 Array Plate. The average of signal intensities of Cy3:Cy5 of each spot was obtained and the ratio >1.5 or <0.67 was defined as the cut-off benchmark to determine the up-regulated or down-regulated genes. Gene ontology analysis was performed to determine the most valuable genes [22].

### Statistical analysis

Observational data were analyzed using the χ2 test for association of categorical data. Spearman's rank correlation was used to examine the concordance between serum CA199 level and PRR11 or among PRR11, UCHL1, SNAT1, and EGR1IHC status. Kaplan-Meier survival analysis was used to determine the time to recurrence and overall survival (OS), with the log-rank test performed to compare survival differences between groups. Cox proportional hazards model for multivariate survival analysis was used to assess predictors related to recurrence and survival. The significance of the *in vivo* data was determined using a two-tailed Mann-Whitney U test. In all tests, a *p* value of less than 0.05 was considered statistically significant. SPSS statistical software and GraphPad Prism 5.0 software were used to perform analyses [21].

## SUPPLEMENTARY FIGURES AND TABLES


