# Supplementary material for: The prognostic potential and oncogenic effects of PRR11 expression in hilar cholangiocarcinoma
Source: Oncotarget. 2015 May 4;6(24):20419–33. doi: 10.18632/oncotarget.3983 (PMC4653015; doi:10.18632/oncotarget.3983)
Supplement: Supplementary file 1 [file oncotarget-06-20419-s001.pdf]

The prognostic potential and oncogenic effects of PRR11 expression in hilar cholangiocarcinoma

Supplementary Material

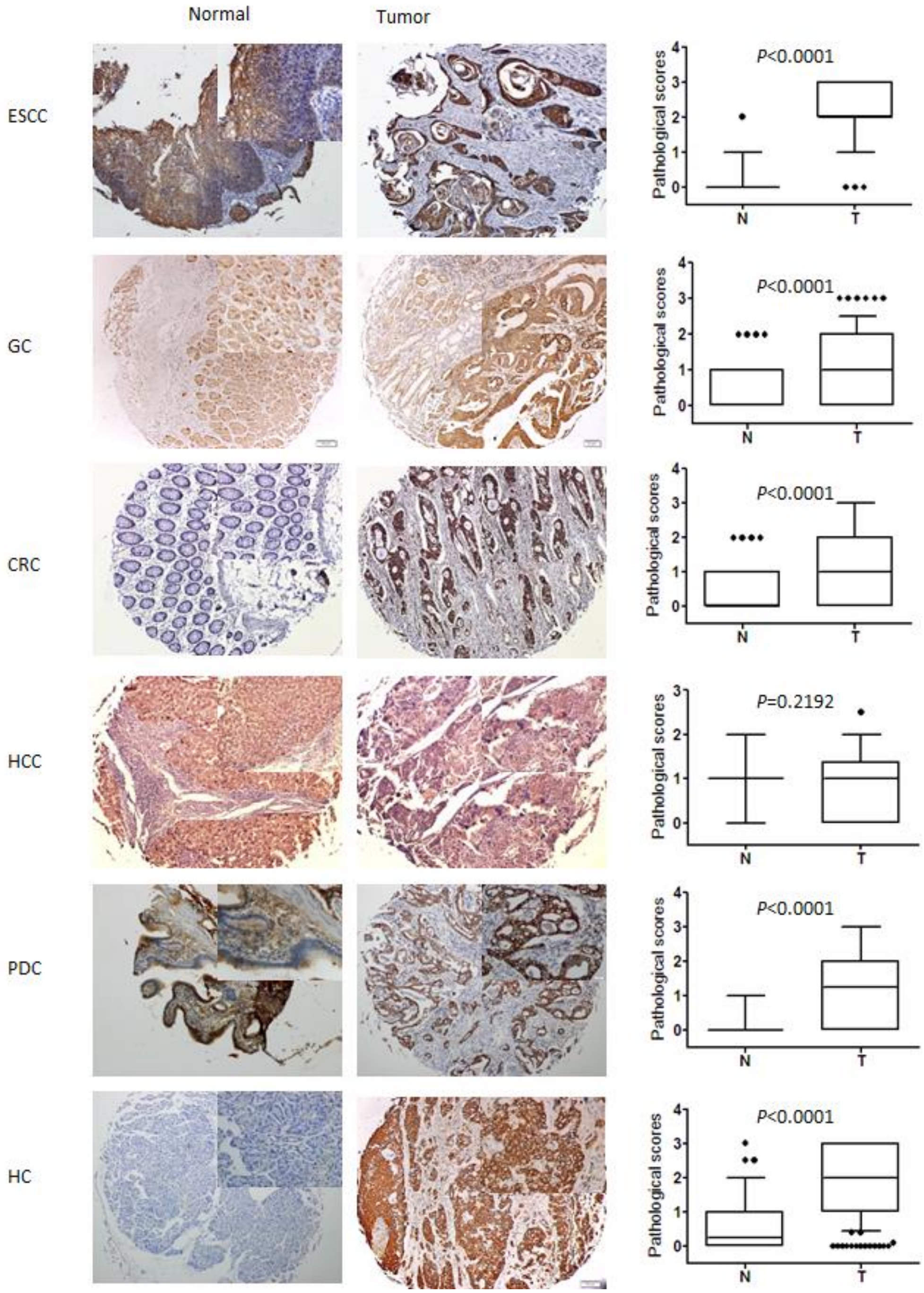

Fig.S1: Expression of PRR11 in the six gastrointestinal tumors and matched non-cancerous tissues. Left, normal tissues; Middle, tumors; Right, Graphical representation of the intensity of PRR11 staining between normal and cancer tissues.

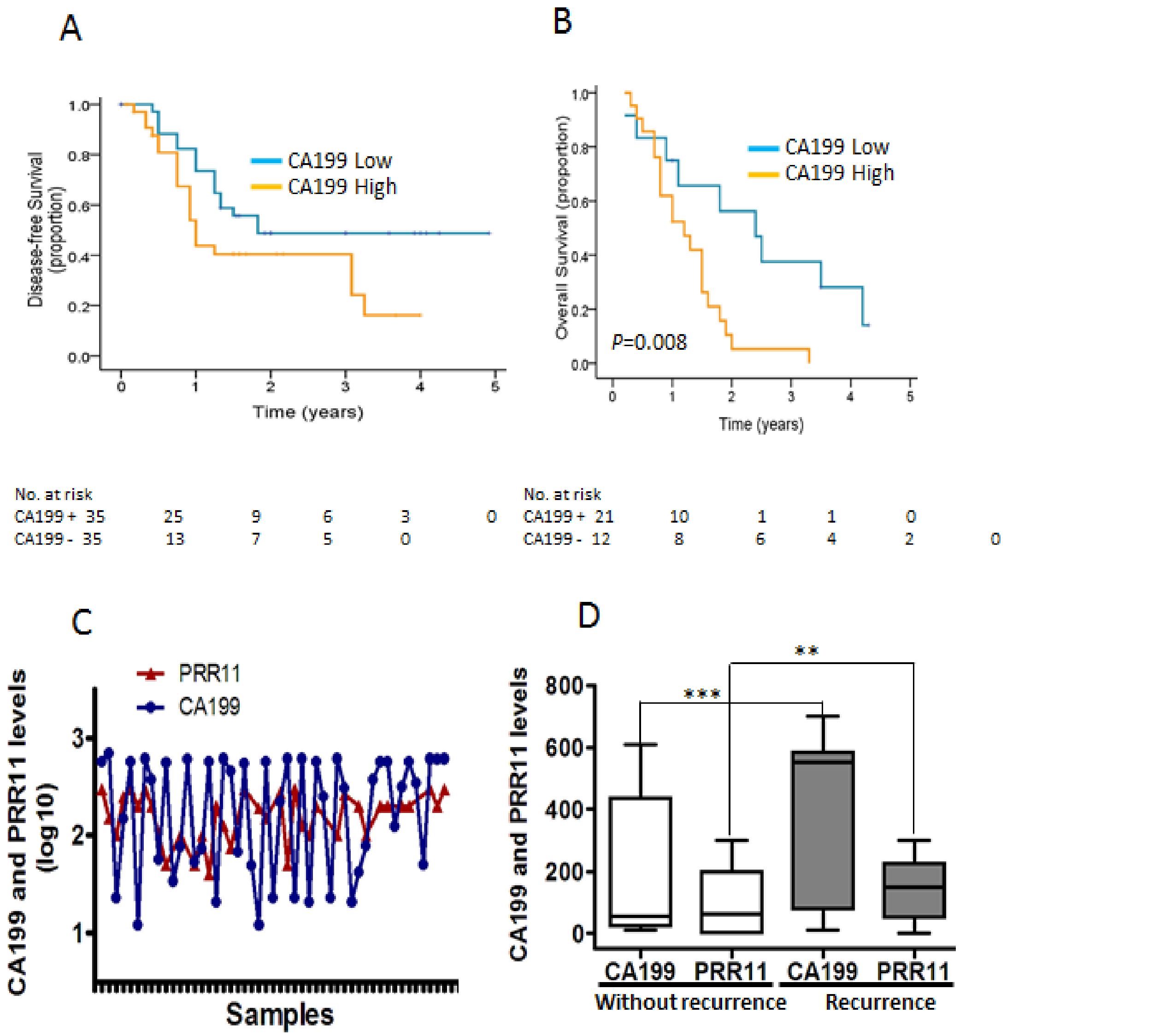

Fig.S2. CA199 levels were associated with disease-free survival (A) and overall survival of patients with hilar cholangiocarcinoma (B). PRR11 levels correlated remarkably with CA199 levels in representative samples (C) and in sub-groups according to recurrence (D). ***P*<0.01, ****P*<0.01.

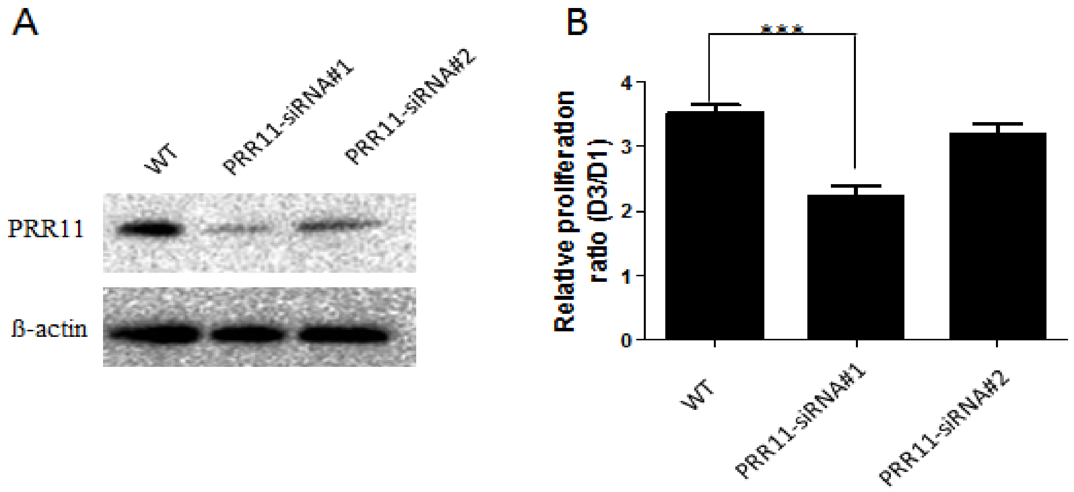

Fig.S3: PRR11-siRNA leads to the inhibition of cell proliferation. Two siRNA (#1 TTC CAG GAG TGA TTA AGA C and #2 AGA CCA TAC TCT ATT TGT C) targeting PRR11 was constructed by Genechem Biotech, Shanghai, China and was transiently transfected into QBC939 cells. Western blot was used to detect the levels of PRR11 protein (A) and MTT assay was used to measure the proliferation ratio between these groups (B).

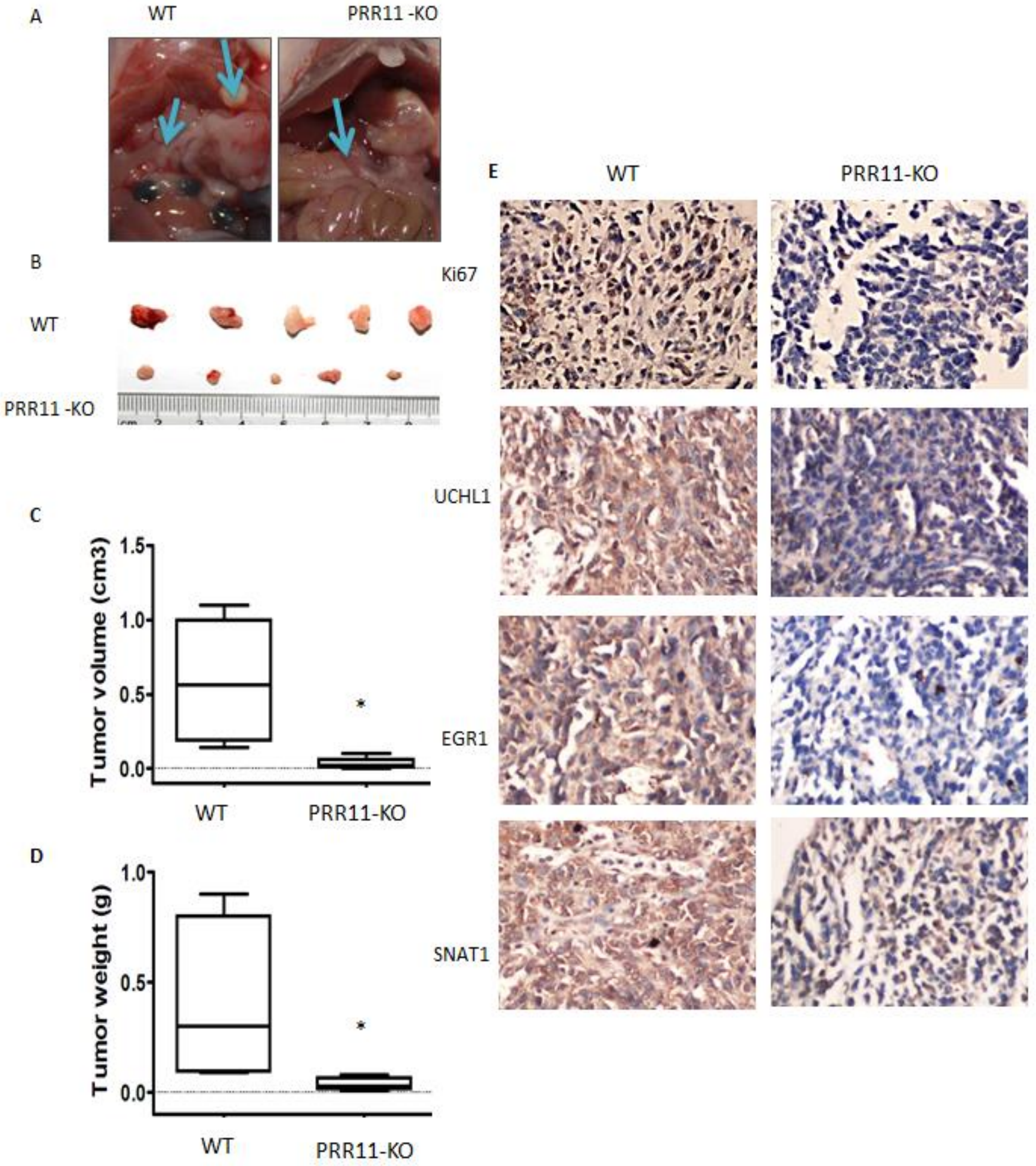

Fig.S4: Representative anatomical pictures (A) and resected tumors (B) of *in situ* tumor transplantation of hilar cholangiocarcinoma cells. The tumor size (D) and tumor weight (E) were evaluated at the end of the experiment. **P*<0.01, compared with the WT group. E) The expression of SNAT1, UCHL1, and EGR1 proteins was detected in resected tumors form the WT group (left) and the PRR11-KO group (right). IHC×400.

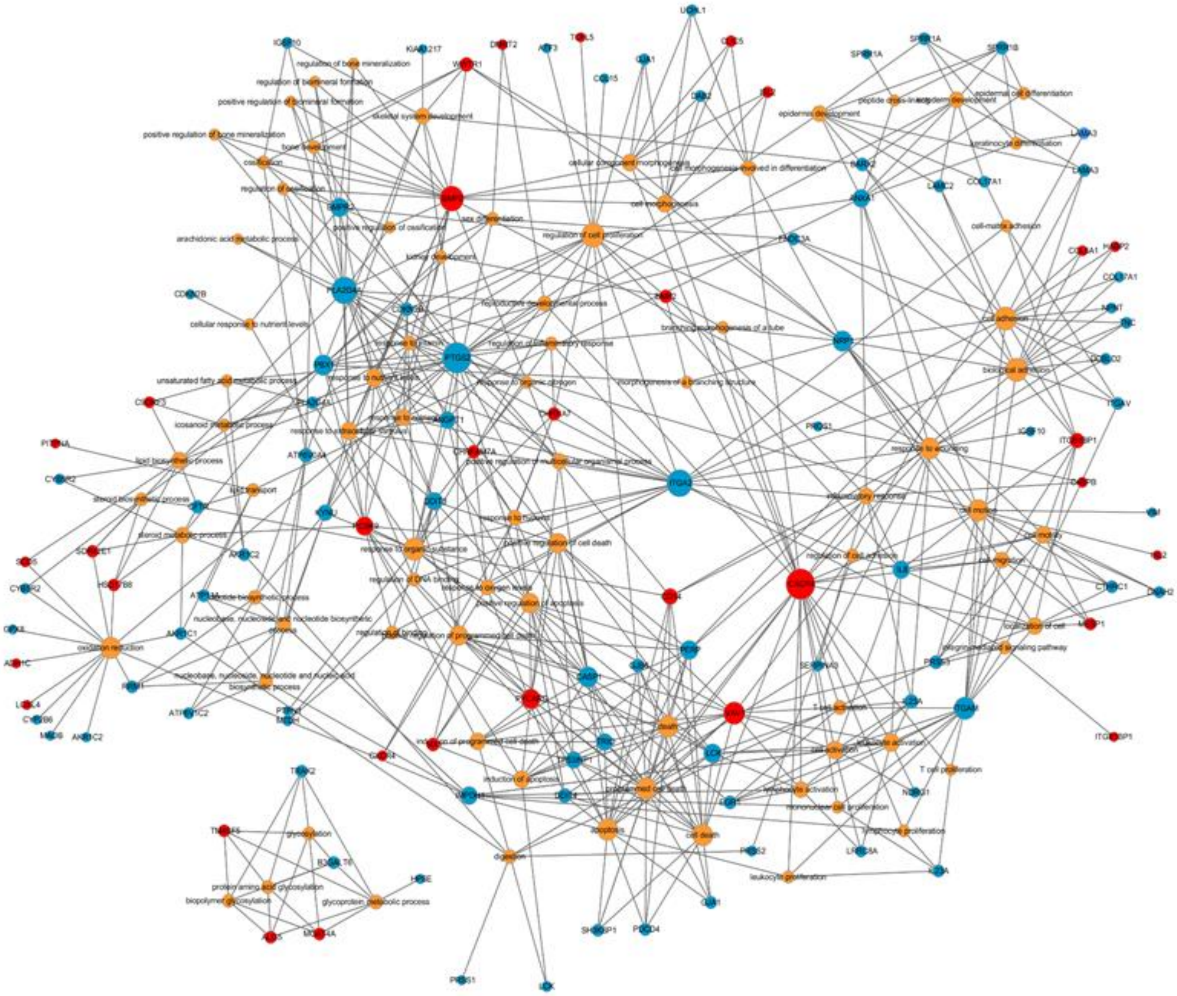

Fig.S5: The correlation between the altered genes and the biological processes analyzed by go-network analysis.

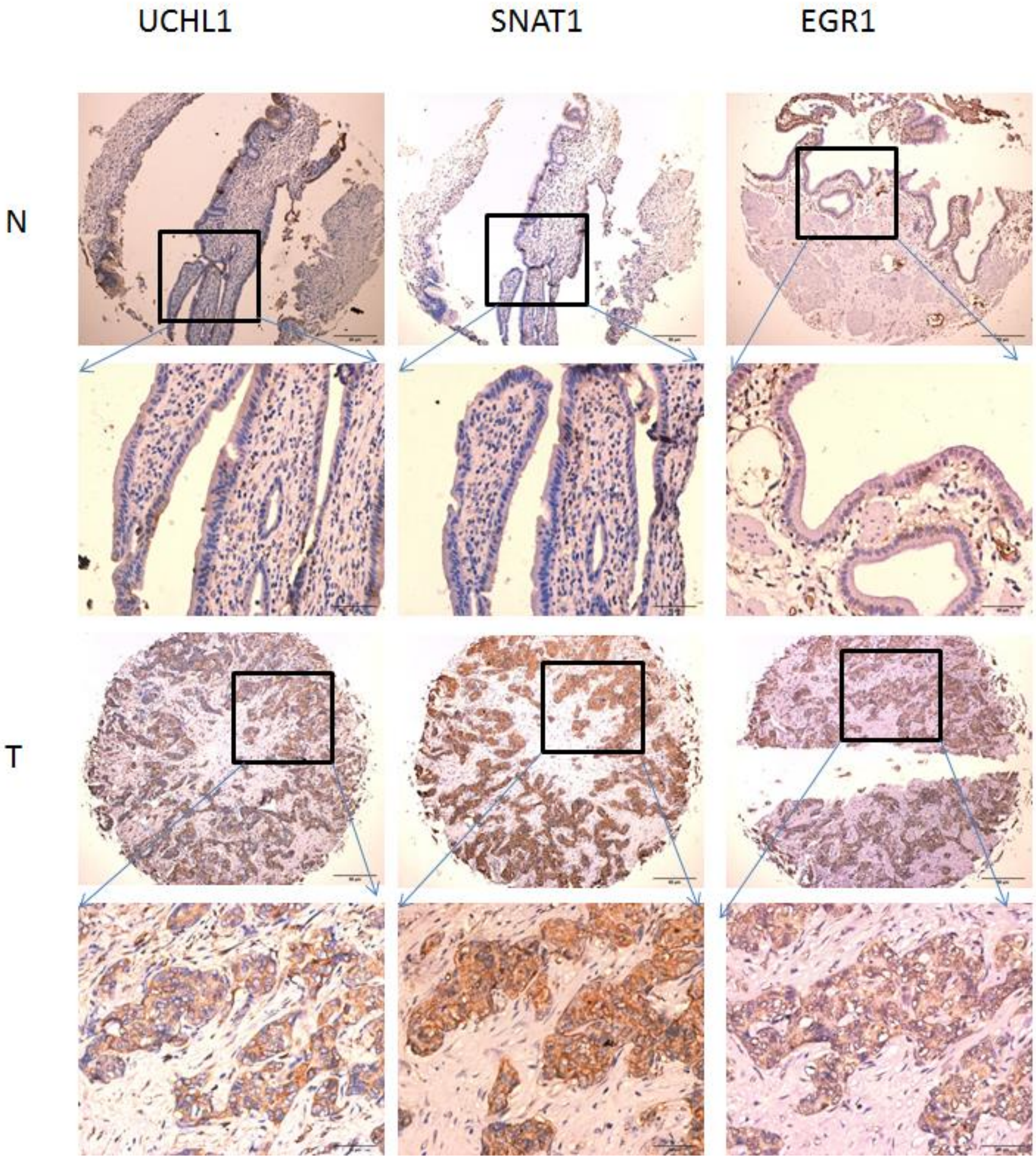

Fig.S6: Analysis of UCHL1, SNAT1, and EGR1 expression in human hilar cholangiocarcinoma. N, normal tissues; T, Tumor samples.

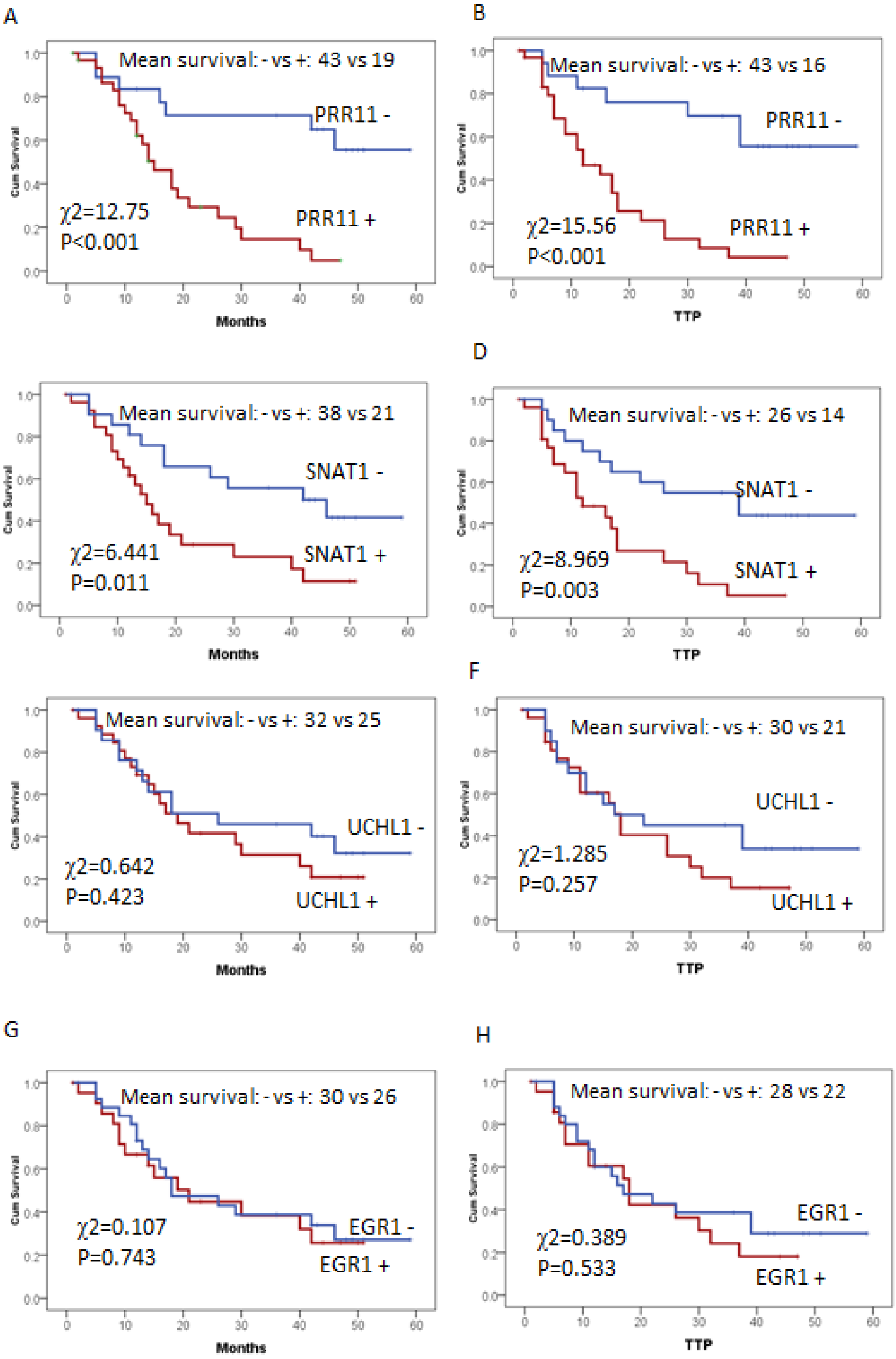

Fig.S7. Survival analysis of the 49 cohort revealed that PRR11 (A, B) and SNAT1 (C, D) is an important predictor for tumor recurrence and prognostic factor for HC patients, while UCHL1 (E, F) and EGR1 (G, H) was not a significant indicator of tumor recurrence and prognosis.

Supplemental Table 1. Correlations between PRR11 expression and CA199 status and levels

PRR11	CA199 status		r	*P*	CA199 level		r	*P*
	Negative	Positive			Low	High		
Negative	10	16	0.230	0.018	18	8	0.259	0.008
Positive	13	66			31	48		

Supplemental Table 2. Univariate and Multivariate Analysis of Variables Associated With OS in Patients Submitted to an R1/2 Resection

Variable	No. of Patients	Median Survival (years)		*P* (univariate)	*P* (multivariate)	Hazard Ratio	95% CI
		Median	Range				
PRR11							
Negative	10	1.80	0.4-4.3	0.023	0.306	0.546	0.172 to 1.736
Positive	24	1.10	0.2-3.3				
CA199 levels							
Low	12	2.40	0.2-4.3	0.008	0.115	0.430	0.150 to 1.228
High	21	1.20	0.3-3.3				

Supplemental Table 3. Association between expression of UCHL1, SNAT1, and EGR1 and clinicopathlogical parameters of hilar cholangiocarcinoma

Parameters	n	UCHL1 positive(%)	*P*	SNAT1 positive(%)	*P*	EGR1 positive(%)	*P*
Age							
≤55y	25	15(60.0)	0.482	14(56.0)	0.897	10(40.0)	0.482
>55y	24	12(50.0)		13(54.2)		12(50.0)	
Gender							
Male	35	18(51.4)	0.414	19(54.3)	0.856	15(42.9)	0.65
Femal	14	9(6.3)		8(57.1)		7(50.0)	
Tumor size							
≤6cm	18	9(50.0)	0.584	9(50.0)	0.584	6(33.3)	0.215
>6cm	31	18(58.1)		18(58.1)		16(51.6)	
T stage							
T1/2	7	2(28.6)	0.127	2(28.6)	0.127	2(28.6)	0.348
T3/4	42	25(59.5)		25(59.5)		20(47.6)	
N stage							
No	16	6(37.5)	0.078	4(25.0)	0.003	3(18.8)	0.010
Yes	33	21(63.6)		23(69.7)		19(57.6)	
Differentiation							
High/moderate	37	20(54.1)	0.796	18(48.6)	0.111	15(40.5)	0.282
Poor	12	7(58.3)		9(75.0)		7(58.3)	

Supplemental Table 4. Data of PRR11 staining

Case Num.	PRR11 staining		
	Raw data	neg. or pos.	intensity
1	1	1	1
2	1.4	1	1
3	2	1	2
4	2	1	1
5	0	0	0
6	1.5	1	1
7	2	1	2
8	2.5	1	3
9	2.5	1	3
10	1.6	1	2
11	2	1	2
12	1	1	1
13	2	1	3
14	3	1	3
15	1	1	1
16	1.5	1	1
17	2	1	1
18	3	1	3
19	1.5	1	1
20	2.5	1	3
21	1	1	1
22	1	1	1
23	3	1	3
24	2	1	2
25	0	0	0
26	2	1	2
27	1.5	1	1
28	1	1	1
29	3	1	3
30	2	1	1
31	2.8	1	3
32	1.5	1	1
33	0.55	0	0
34	0	0	0
35	1	1	2
36	1	1	1
37	3	1	3
38	1	1	1
39	0.6	0	0
40	0	0	0
41	2	1	2
42	2.5	1	3
43	0	0	0

44	3	1	3
45	3	1	3
46	2	1	2
47	3	1	3
48	3	1	3
49	3	1	3
50	2	1	2
51	1	1	3
52	1	1	3
53	0	0	0
54	1	1	1
55	1.8	1	2
56	2	1	2
57	2	1	2
58	2.2	1	2
59	3	1	3
60	1	1	1
61	2	1	2
62	3	1	3
63	2.8	1	3
64	2.4	1	2
65	3	1	3
66	1	1	1
67	0	0	0
68	3	1	3
69	0	0	0
70	1	1	1
71	0	0	0
72	0	0	0
73	2.2	1	2
74	0	0	0
75	2	1	2
76	0.5	1	1
77	1	1	1
78	0	0	0
79	1	1	3
80	0	0	0
81	0	0	0
82	2.2	1	2
83	0	0	0
84	3	1	3
85	0	0	0
86	1	1	3
87	1	1	2
88	0	0	0
89	1	1	2
90	0	0	0

91	0	0	0
92	0.8	1	1
93	3	1	3
94	0	0	0
95	0.5	1	1
96	1	1	1
97	0	0	0
98	2.3	1	2
99	1.8	1	2
100	0	0	0
101	2	1	2
102	1	1	1
103	0	0	0
104	0	0	0
105	3	1	3
106	2.2	1	2
107	3	1	3
